# Temporal trend and magnitude of previdenciary benefits for workplace accidents in Brazil

**DOI:** 10.1590/1980-549720240032

**Published:** 2024-06-24

**Authors:** Claudio José dos Santos, Frida Marina Fischer

**Affiliations:** I Universidade de São Paulo, Faculty of Public Health – São Paulo (SP), Brazil.

**Keywords:** Workplace accidents, Occupational injuries, Workplace accidents registry, Social security, Time series studies, Acidentes do trabalho, Lesões ocupacionais, Registro de acidentes do trabalho, Seguro social, Estudos de séries temporais

## Abstract

**Objective:**

To analyze the temporal trend and magnitude of national indicators of previdenciary benefits for workplace accidents issued and granted by the Social Security of Brazil.

**Methods:**

Secondary data from Social Security from 2008 to 2019 were used. The trend and percentage variation of the indicators were estimated through Prais-Winsten generalized linear regression.

**Results:**

A total of 9,220,372 previdenciary benefits for workplace accidents were issued by the Social Security of Brazil in the period, costing approximately R$ 8.4 billion and representing about 2.0% of the net value of all benefits paid. None of the categories of previdenciary benefits for workplace accidents showed an increasing trend. The highest variation in the benefits granted and issued for workplace accidents occurred in temporary disability benefit (B91), with an annual percentage variation of -54.00% and -29.29%, respectively.

**Conclusion:**

A reduction in magnitude and an overall decreasing trend were observed in the historical series of national indicators of benefits granted and benefits issued related to workplace accidents in Brazil from 2008 to 2019.

## RESUMO

**Objetivo:**

Analisar a tendência temporal e a magnitude dos indicadores nacionais de benefícios previdenciários emitidos e concedidos por acidentes do trabalho pela Previdência Social do Brasil.

**Métodos:**

Foram utilizados dados secundários da Previdência Social de 2008 a 2019. A tendência e variação percentual dos indicadores foram estimadas por meio de regressão linear generalizada de Prais-Winsten.

**Resultados:**

Um total de 9.220.372 benefícios previdenciários por acidentes do trabalho foi emitido pela Previdência Social do Brasil no período, custando aproximadamente R$ 8,4 bilhões e representando cerca de 2,0% do valor líquido de todos os benefícios pagos. Nenhuma das categorias de incapacidades relacionadas a acidentes do trabalho apresentou tendência de aumento. A maior variação nos benefícios concedidos e emitidos por acidentes do trabalho ocorreu no auxílio por incapacidade temporária (B91), com variação percentual anual de -29,29% e -54,00%, nessa ordem.

**Conclusão:**

Verificou-se redução na magnitude e tendência global decrescente em relação as séries históricas dos indicadores nacionais de benefícios concedidos e emitidos de natureza acidentária no Brasil, de 2008 a 2019.

## INTRODUCTION

Workplace accidents and injuries present a significant occupational health concern both in Brazil and in the global context^1^. The International Labour Organization (ILO) estimates that approximately 374 million non-fatal injuries occur among workers worldwide each year, with 7,500 people dying daily due to work-related conditions. Out of these fatalities, 6,500 are attributed to occupational diseases, and 1,000 to workplace accidents^2^.

In the Brazilian context, recent studies have shown a trend toward reducing workplace accidents. However, the incidence of these events remains high^3,4^. Research analyzing contributors to the General Social Security System (RGPS) revealed a long-term trend of decrease in typical accidents, occupational diseases, and total accidents between 2008 and 2013^3^. In line with this, Bezerra et al. demonstrated that from 2008 to 2014, the incidence of these events decreased by 29.69%, with rates declining from 14.01 to 9.85 per 1,000 workers, respectively^4^. Despite these notable figures, they do not fully capture the actual number of workplace accidents due to underreporting^5^.

Such occurrences bring significant consequences for workers, their families, the state, and society at large. These implications range from income loss and dependency on external assistance for daily tasks to substantial increases in financial burdens related to social security benefits, including sickness benefits, disability retirement, death pensions, and life insurance. Additionally, there are expenses related to labor lawsuits, compensations, and medical costs such as examinations, hospitalizations, surgeries, medications, and rehabilitation treatments^6^.

It is estimated that workplace accidents result in an economic impact equivalent to around 4% of the global Gross Domestic Product (GDP), surpassing an annual total of USD $1.25 trillion^7,8^. In the Brazilian context, this percentage translates to approximately R$ 396 billion per year, considering Brazil’s GDP in 2022, which reached R$ 9.9 trillion^9^. Furthermore, workplace accidents lead to decreased tax revenue due to incapacitated or deceased workers no longer contributing to the social security system. Additionally, they contribute to increased poverty and social exclusion, while also overburdening healthcare and social assistance systems^10^.

Despite progress in recording workplace accidents in the past decade, along with efforts in occupational health, safety initiatives, prevention, and worker health monitoring, and increased accessibility to data on such incidents^11^, studies examining trends in the costs of workplace accidents over time are limited or, if present, outdated.

This current study aimed to analyze the temporal trend and magnitude of national indicators of previdenciary benefits for workplace accidents issued and granted by the Social Security of Brazil.

## METHODS

This study employed an ecological time series analysis focusing on national indicators of Workplace Accident Benefits Issued (WABII) and Granted (WABGI) by the Brazilian Social Security Institute (INSS) to beneficiaries of the RGPS.

The data are from the Single Benefit Information System (SUIBE), and were extracted through the Social Security Statistical Yearbook — AEPS InfoLogo (http://www3.dataprev.gov.br/infologo/) covering the period from 2008 to 2019. The selected timeframe aimed to minimize the impact of the implementation of the Epidemiological Technical Nexus (NTEP) by the INSS in April 2007, which led to substantial variations in reported workplace accidents in Brazil. Thus, the initial months of NTEP implementation in 2007 were excluded from the analysis. Selecting 2019 as the end of the historical series aimed to avoid potential interference from the COVID-19 pandemic on workplace accidents indicators.

Outcome variables comprised WABII and WABGI along with the proportion of net credits issued in national currency corresponding to accident-related benefits relative to all RGPS benefits for the year.

Calculations were as follows:

a) WABII = Number of benefits issued in the year/Number of RGPS beneficiaries for the year x 1,000;
b) WABGI = Number of benefits granted in the year/Number of RGPS beneficiaries for the year x 1,000;
c) Proportion = Value in R$ corresponding to accident-related benefits issued in the year/Value in R$ of all RGPS benefits issued in the year x 100.

The definitions of benefits granted and benefits issued were derived from the Social Security Statistical Bulletin:

a) Benefits issued: These refer to credits sent to banking networks for the payment of ongoing benefits listed in the active registry. In essence, these are the benefits paid monthly that appear in the INSS’s monthly payroll;
b) Benefits granted: Correspond to benefit applications submitted by beneficiaries to the Social Security of Brazil, reviewed, approved, and released for payment due to satisfying all necessary criteria for the requested benefit^12^.

The indicators were computed for the four previdenciary benefits for workplace accidents stipulated by the Social Security Benefit Plan in effect in Brazil:

a) temporary disability benefits due to workplace accident, formerly known as “sickness benefits” (Code B91);
b) “disability retirement” due to workplace accident (Code B92);
c) “death pension” due to workplace accident (Code B93); and
d) “accident benefit” due to workplace accident (Code B94)^13^.

Temporal trend analysis followed the methodological recommendations of Antunes and Cardoso^14^. Annual percentage change (APC) and 95% confidence intervals (CI) were estimated with a significance level of 5% using Prais-Winsten generalized linear regression, which corrects for first-order autocorrelation in time series analysis^15^. Trends were categorized as “increase” (APC>0; p<0.050), “decrease” (APC<0; p<0.050) or did not show a trend, according to the model adopted (“stable trend”) (p>0.050)^14^. All analyses were conducted using Stata 17.1 Software (College Station, TX, USA, 2021).

This study was based on open-access and public domain data, thus ethical committee approval was not required.

## RESULTS

A total of 9,220,372 workplace accident-related benefits were issued by the Social Security of Brazil between 2008 and 2019, with 3,580,683 benefits granted to beneficiaries. Over the twelve-year study period, these benefits incurred a cost of approximately R$ 8.40 billion to the Social Security funds, representing around 2.0% of the net values of credits issued by the agency for social security benefit payments.

The analysis of the annual averages of benefits issued per workplace accident-related category unveiled that accident benefits had an annual average of 305,758 benefits, followed by 189,048 disability retirements, 154,960 temporary disability benefits, and 118,599 death pensions. Regarding granted benefits, the annual averages were 270,037 for temporary disability benefits, 17,586 for accident benefits, 10,220 for disability retirements, and 548 for death pensions. These findings are summarized in Table 1, presenting the absolute numbers of workplace accident-related benefits issued and granted by the Social Security of Brazil during the analyzed period.

**Table 1.** Quantity of workplace accident-related benefits issued and granted by the Social Security of Brazil, by categories. Brazil, 2008–2019.

Year	Temporary disability (B91)		Disability retirement (B92)		Death pension (B93)		Accident benefit (B94)	
	Issued	Granted	Issued	Granted	Issued	Granted	Issued	Granted
2008	170,654	356,336	153,260	7,839	127,985	1,127	272,511	11,538
2009	161,541	329,914	159,766	8,940	126,740	850	275,448	13,472
2010	183,330	327,894	166,339	10,261	125,391	778	281,058	12,655
2011	179,098	319,445	173,759	11,108	123,930	751	288,673	15,068
2012	175,145	305,208	181,599	11,433	122,331	614	295,318	16,012
2013	182,030	304,217	189,161	11,655	120,606	497	304,373	21,563
2014	175,135	279,868	196,175	10,877	118,543	412	312,796	20,883
2015	155,606	196,761	201,124	8,782	116,339	368	317,664	16,399
2016	152,160	228,151	206,171	9,572	114,045	401	322,182	19,055
2017	123,345	195,179	210,877	9,519	111,688	305	326,271	20,253
2018	107,565	202,406	215,092	11,372	108,981	212	332,846	21,281
2019	93,912	195,059	215,252	11,281	106,603	260	339,954	22,852
**Average**	154,960	270,037	189,048	10,220	118,599	548	305,758	17,586
**Total**	1,859,521	3,240,438	2,268,575	122,639	1,423,182	6,575	3,669,094	211,031

Table 2 outlines the trends and APC of WABII and WABGI per category. Temporary disability benefits in Brazil displayed a declining trend in both WABII and WABGI from 2008 to 2019. The WABII exhibited a significant decline (APC: -29.29%; 95%CI -34.19 to -24.03), and the same behavior was presented by the WABGI (APC: -54.0%; 95%CI -62.92 to -42.94), signifying a notable reduction in the rate of benefits issued and granted during the period studied.

**Table 2.** Trend and annual percentage change in the benefits issued and benefits granted for workplace accidents (both per 1,000 contributors), by categories. Brazil, 2008–2019.

Year	Temporary disability (B91)		Disability retirement (B92)		Death pension (B93)		Accident benefit (B94)	
	WABII	WABGI	WABII	WABGI	WABII	WABGI	WABII	WABGI
2008	3.16	6.60	2.84	0.15	2.37	0.02	5.05	0.21
2009	2.89	5.90	2.86	0.16	2.27	0.02	4.93	0.24
2010	3.05	5.45	2.76	0.17	2.08	0.01	4.67	0.21
2011	2.79	4.98	2.71	0.17	1.93	0.01	4.50	0.24
2012	2.60	4.54	2.70	0.17	1.82	0.01	4.39	0.24
2013	2.61	4.36	2.71	0.17	1.73	0.01	4.36	0.31
2014	2.45	3.92	2.75	0.15	1.66	0.01	4.38	0.29
2015	2.23	2.83	2.89	0.**1**3	1.67	0.01	4.56	0.24
2016	2.28	3.42	3.09	0.14	1.71	0.01	4.83	0.29
2017	1.89	2.99	3.23	0.15	1.71	0.00	5.00	0.31
2018	1.64	3.09	3.28	0.17	1.66	0.00	5.08	0.32
2019	1.40	2.91	3.21	0.17	1.59	0.00	5.07	0.34
**Average**	2.39	4.17	2.92	0.16	1.83	0.01	4.72	0.27
**APC (%)**	-29.29	-54.00	8.76	0.16	-14.97	-0.34	0.75	2.51
**95CI%**	-34.19; -24.03	-62.92; -42.94	-1.40; 19.98	-0,82;0.14	-21.73; -7.64	-0.48; -0.19	-17.44; 22.96	1.44; 3.58
**p-value**	<0.001	<0.001	0.086	0.731	0.001	<0.001	0.935	<0.001
**Trend**	↓	↓	↑↓	↑↓	↓	↓	↑↓	↓

**↑: increase;** ↓**: decrease; ↑**↓**: without linear trend; WABII: Workplace Accident Benefits Issued Indicator; WABGI: Workplace Accident Benefits Granted Indicator; APC: annual percentage change; CI: confidence interval.**

Death pensions experienced a decreasing trend in both WABII (APC: -14.97%; 95%CI -21.73 to -7.64) and WABGI (APC: -0.34%; 95%CI -0.48 to -0.19). Accident benefits (B94) in Brazil displayed a stable trend in WABII and a significant decreasing trend in WABGI (APC: 2.51%; 95%CI 1.44 to 3.58). Disability retirement benefits did not show a tendency toward the method. The slight increase in WABII of disability retirement benefits was not statistically significant, and the WABGI showed little variation over the analyzed period.

Among the four workplace accident-related categories, the largest resources were allocated to disability retirement payments, which exhibited a stable trend in the proportion of credits issued relative to the net value of all RGPS benefits. This same lack of trend behavior was presented by the accident benefit proportion. In contrast, the other workplace accident-related benefit categories showed a trend of decreasing percentages in relation to the value of credits issued by the RGPS, as presented in Table 3.

**Table 3.** Net value of credits issued for social security workplace accident-related benefits, by categories; trend and annual percentage change of percentages in relation to net value of social security benefits. Brazil, 2008–2019.

Year	RGPS benefits		Temporary disability (B91)		Disability retirement (B92)		Death pension (B93)		Accident benefit (B94)	
	R$^1^	R$^2^	%	R$^2^	%	R$^2^	%	R$^2^	%
2008	13.82	127.66	0.92	112.28	0.81	82.23	0.60	102.09	0.74
2009	15.48	126.34	0.82	125.78	0.81	87.55	0.57	112.91	0.73
2010	17.43	154.11	0.88	142.23	0.82	93.66	0.54	126.55	0.73
2011	19.08	157.34	0.82	158.17	0.83	97.90	0.51	141.02	0.74
2012	21.62	170.30	0.79	178.00	0.82	104.43	0.48	157.39	0.73
2013	24.29	190.66	0.78	201.20	0.83	111.32	0.46	177.49	0.73
2014	26.68	196.08	0.73	221.77	0.83	116.54	0.44	196.17	0.74
2015	29.24	192.10	0.66	241.45	0.83	121.74	0.42	214.92	0.74
2016	33.88	206.79	0.61	276.61	0.82	132.63	0.39	245.05	0.72
2017	37.00	176.65	0.48	302.15	0.82	138.74	0.37	265.58	0.72
2018	38.44	159.14	0.41	311.40	0.81	138.12	0.36	279.25	0.73
2019	41.09	145.60	0.35	324.10	0.79	141.57	0.34	298.59	0.73
**APC (%)**			-11.06		-0.35		-5.29		-0.19
**95CI%**			-14.32; -7.67		-1.26; 0.57		-5.93; -4.65		-0.49;0.10
**p-value**			<0.001		0.420		<0.001		0.180
**Trend**			↓		↑↓		↓		↑↓

^1^ **values expressed in billions of reais;** ^2^ **values expressed in millions of reais; %: percentage relative to net value of benefits issued by Social Security of Brazil for the year; ↑: increase;** ↓**: decrease; ↑**↓**: without linear trend; RGPS: General Social Security System; APC: annual percentage change; CI: confidence interval.**

## DISCUSSION

In the analysis of temporal trends in the WABGI and WABII, obtained through Prais-Winsten linear regression, none of the four included social security categories (B91, B92, B93, B94) showed an increasing trend. Overall, the historical series of issuance or granting of death pension, disability assistance, disability retirement, and accident benefit due to workplace accident shows trends of reduction or stability (understood here as the absence of trend according to the employed method).

The highest variation in the granting of types of workplace accident-related benefits occurred in temporary disability assistance (B91), with an annual percentage variation of -54.00%. This same benefit also showed the highest negative annual percentage variation in WABII (-29.29%). Additionally, there was a reduction trend in the granting and issuance of accident-related death pension (B93) and in the granting of accident benefit due to workplace accident (B94).

The overall reduction in the quantity of issued and granted benefits can be interpreted as a potential improvement in workplace safety and health over the years. The decreasing trend in indicators of costs related to occupational beneficiaries, in this logic, may reflect a potential outcome of government policies and programs aimed at safer and healthier work environments, such as the National Network for Comprehensive Worker Health Care (Renast)^16^, the National Policy and Plan for Safety and Health at Work (PLANSAT and PNSST)^17^, Accident Prevention Factor (FAP)^18,19^, NTEP^20^, and National Health Policies for Male and Female Workers (PNSTT)^21,22^. These measures — despite contradictions, limitations, and challenges/opportunities that still exist — drive improvements in health and safety conditions at the national level, contributing to safer and healthier workplaces and the reduction of workplace accident indicators.

Recently published studies have demonstrated a persistent decline in reported workplace accidents in the country^3,4,18,19,23-25^, a finding consistent with the decrease in the granting/issuance of social security benefits for workplace accidents identified in the present research.

Few studies monitoring, evaluating, and measuring the impact of public policies on workplace accident rates have been identified in Brazil. Among the scarce initiatives aimed at this purpose, notable are the studies conducted by Wernke et al.^18^ and Shimizu et al.^19^. In the study conducted by Wernke et al., a comprehensive analysis of workplace accident rate series revealed a notable shift in patterns. Prior to the implementation of the FAP methodology, between 2006 and 2009, the research identified an indefinite trend in the evolution of these rates. However, a significant transformation was observed during the FAP implementation from 2010 to 2016, with nearly all analyzed workplace accident rate series demonstrating a notable and statistically significant reduction. While the study acknowledges its methodological limitations and refrains from definitively asserting causality in the results, the logic of inducing more effective investments in occupational health and safety brought by the FAP seems to have played a crucial role in the outcomes^18^. The research conducted by Shimizu et al. also investigated the incidence of workplace accidents/illnesses in Brazil before and after FAP introduction in 2010. Analyzing data from 2008 to 2014 from the Social Security of Brazil, the authors noted an overall reduction in the incidence of workplace accidents/illnesses. This reduction was particularly notable in injuries, poisonings, and other diseases due to external causes, musculoskeletal and connective tissue disorders, mental and behavioral disorders, and diseases of the nervous system. The decrease was observed across all studied groups: manufacturing and production; retail and motor vehicle repair; social services and human health; construction; and transportation, storage, and postal services. However, a significant reduction, following the introduction of the FAP, was only observed in manufacturing and production activities^19^.

Shimizu et al. noted that the FAP addresses the frequency and severity of accidents, but neglects essential aspects related to occupational health and safety. According to the authors, Brazil has failed to match the reduction of workplace accidents observed in industrialized countries, nor has it implemented effective improvements in preventive practices or structural changes in work environments. They argue that the FAP may, actually, result in underreporting of workplace accidents, as employers may choose not to report minor injuries since an increase in the FAP implies higher taxes, thus incentivizing non-reporting. However, they highlight that in more serious cases, such as fatal accidents or those resulting in disabilities — as is the case with those leading to the benefits analyzed here — underreporting is less likely due to the more severe consequences and investigations that typically occur. Thus, for the authors, the FAP appears to have reduced the number of more serious accidents taking into account the financial implications for employers^19^. The establishment of the FAP may have incentivized underreporting by companies, as it brings fiscal benefits to employers.

The reduction in the formalization of the labor market and the growing indicators of informal work in Brazil may also have contributed to the results identified in this research^26,27^. In a recent study, it was found that among the total employed population, informal work accounted for 39.8% in 2012, a number that rose to 43.4% in 2019, the final year of this study. The most recent percentage of informally employed individuals estimated for 2022 was 46.0%, reflecting the increasing prevalence of informality in the Brazilian economy^26^. Informality on the labor market often signifies the absence of labor rights and exposure to precarious conditions, making workers more vulnerable^28,29^. Informal workers, even when affiliated with the RGPS as self-employed/professional freelancers, do not fall under the concept of workplace accidents according to social security law (Article 19 of Law 8,213/1991), and therefore, do not have access to workplace accident-related social security benefits. These professionals, even if they experience accidents in the workplace or during the provision of services, have this condition classified as an accident of any nature^13^.

The disability retirement (B92), a benefit granted to workers permanently incapacitated for performing their duties due to workplace accidents, absorbed the largest share of financial resources allocated to accident benefits. This greater prominence in overall costs compared to other benefits is likely due to the fact that disability retirements are long-term benefits that extend throughout the insured’s lifetime, increasing their cumulative impact on the payroll^30,31^. This same benefit exhibited a stable behavior in the historical series (without a trend of reduction or growth by the method) regarding granting and issuance — a pattern also observed for the issued of accident assistance, another benefit of extended duration due to its compensatory nature and ceasing only upon the insured’s retirement.

The net value of R$ 8.40 billion spent on workplace accident benefits over the twelve analyzed years represented approximately 2.0% of the net values of credits issued for all RGPS benefits between 2008 and 2019. These numbers reflect the importance of workplace accident benefits in the broader context of resources allocated to RGPS benefits, demonstrating the economic burden of workplace accidents for the country. This proportion is relevant because it contributes to assessing the sustainability of the social security system, especially in the context of population aging and demographic changes^32,33^. The distributive objectives of RGPS need to be pursued without disregarding the sustainability of this policy. Thus, mitigating situations that result in new benefit grants is essential to maintain the financial balance of the overall social security and pension system^34^.

It is worth noting that workplace accidents are recognized as events with notorious underreporting in Brazil, resulting in an underestimation of the true incidence of these occurrences in the national territory^10,35^, which may have influenced the results of this study. Another possible element of imprecision in the phenomenon assessed in this work is the non-establishment of a causal link between the injury and work activity, either by the company, which may fail to issue the Workplace Accident Notification (CAT), or by the medical examiner, who may not recognize the NTEP^36^.

It is emphasized that the aim of this study was not to provide a comprehensive list of all costs involving workplace accidents but to present the trend and magnitude of social security benefits related to them, one of the direct costs of workplace accidents in Brazil, based on administrative records. Expanded estimates of direct and indirect costs of accidents were recently published in a study conducted in Brazil^37^.

This study has limitations. One of them is that we confined the analysis only to net social security expenses resulting from workplace accidents recorded by the Social Security of Brazil, covering only RGPS insured individuals, without including expenses related to statutory servants, for example. Furthermore, the variables were not presented in gross values, as these are not provided by the Ministry of Social Security in AEPS InfoLogo. Additionally, we did not analyze the evolution of costs related to labor lawsuits, compensations, medical-hospital expenses, or expenditures on hospitalizations, surgeries, medications, and rehabilitation treatments — information that could provide a comprehensive overview of the magnitude of workplace accidents in the country. Another aspect is that the data cannot be generalized to informal workers, who lack access to accident benefits covered by Social Security of Brazil.

Despite all considerations, the data presented in this study bring significant contributions since there is a shortage of recent research in the Brazilian scientific literature on the temporal trend and magnitude of social security benefits related to workplace accidents. Understanding the dynamics of this phenomenon is crucial for analyzing the role of social security, health, and occupational safety policies in the country and for identifying potential challenges and trends regarding government programs and actions that aim to ensure safer and healthier work environments, as well as mitigate economic impacts within social security expenses.

The results of this study indicated a reduction in magnitude and an overall decreasing trend in national granted and issued indicators of social security benefits related to workplace accidents issued and granted by the Social Security of Brazil from 2008 to 2019. However, the overall numbers of benefits are still significant, reflecting the weight of these occurrences on national Social Security resources. Investing in prevention, training, inspection, and awareness policies, therefore, remains a crucial and necessary measures to further reduce accidents and, consequently, their costs.

Additional studies should be conducted to accurately identify the specific elements and factors that contributed to the reported changes. Conducting impact evaluation research on public policies such as PLANSAT, PNSST, PNSTT, FAP, and NTEP, and assessing the effect of changes in regulations related to benefits granted by the INSS, among others, on workplace accident indicators, including those related to the costs of social security benefits, could provide a comprehensive understanding of the factors involved in the identified changes in magnitude and trend, as well as guide future occupational health and safety policies and facilitate the improvement of existing ones.
